# MEPED as salvage therapy for relapsed/refractory Hodgkin’s lymphoma incorporating edited non-oncogene addiction: mTOR as a bottleneck

**DOI:** 10.3389/fphar.2025.1553331

**Published:** 2025-03-20

**Authors:** Dennis Christoph Harrer, Florian Lüke, Tobias Pukrop, Lina Ghibelli, Albrecht Reichle, Daniel Heudobler

**Affiliations:** ^1^ Department of Internal Medicine III, Hematology and Oncology, University Hospital Regensburg, Regensburg, Germany; ^2^ Division of Personalized Tumor Therapy, Fraunhofer Institute for Toxicology and Experimental Medicine, Regensburg, Germany; ^3^ Bavarian Cancer Research Center (BZKF), University Hospital Regensburg, Regensburg, Germany; ^4^ Department of Biology, University of Rome “Tor Vergata”, Rome, Italy

**Keywords:** relapsed/refractory Hodgkin’s lymphoma, non-oncogene addiction, mTOR, M-CRAC, Hodgkin’s lymphoma tissue editing, anakoinosis, pioglitazone, dexamethasone

## Abstract

Rescue therapies of relapsed/refractory (r/r) Hodgkin’s lymphoma (HL) in the third to sixth-line provide major, yet unresolved problems. The MEPED regimen includes nuclear receptor agonists such as pioglitazone and dexamethasone, which counterbalance HL homeostasis, HL stress response inhibitors, everolimus and COX-2 inhibitor, and a stress response inducer, low-dose metronomic treosulfan. CR (six of seven patients) and long-term cCR in patients receiving no consolidating allogeneic stem cell transplantation highlight MEPED as a potent salvage therapy in advanced refractory HL. MEPED edits everolimus activities in such a way that mTORC1 becomes a non-oncogene addiction bottleneck, hence determining long-term therapy outcome. The implications of the therapeutic paradigm shift toward editing of HL tissue, and particularly mTOR addiction, could prove to be profound for clinical practice, both in terms of outcome and treatment tolerability. The long-term results of MEPED treatment indicate the urgent evaluation of the schedule in a multicenter trial for r/r HL.

## Introduction

The incidence of Hodgkin’s lymphomas (HLs) in 2020 was 0.4% for all newly reported cancer-related cases, and 0.2% of deaths were due to Hodgkin’ lymphoma (Huang et al., 2022). The current first-line treatment results for HL are outstanding. The 5-year event-free survival (EFS) is 79.1% for children with stage IIIB or IVB disease, with a high rate of involved-site radiation therapy (76%). An OS between 89% and 94% at 6 years has been observed for adult patients (Kelly and Friedberg, 2024). Nevertheless, 10 to 30% of patients show relapsing or refractory disease, highlighting the urgent quest to identify potent salvage therapies for those patients (Ansell, 2020; Grover et al., 2023). Salvage therapies are dose-intensive and include chemotherapy followed by high-dose chemotherapy with autologous stem cell transplantation (ASCT), targeted chemotherapy with brentuximab vedotin (BV), and immune checkpoint inhibitors (ICPi) such as nivolumab or pembrolizumab (Cellini et al., 2023). Allogeneic stem cell transplantation (allo-SCT) serves as a non-standard treatment but has a curative role in selected cases of relapsed/refractory (r/r) HL (Sureda et al., 2012; Mehta-Shah and Bartlett, 2018). Beyond these salvage treatments, no further standard therapy options exist for r/r HL, with particularly very few curative options (Chen et al., 2019; Kuruvilla et al., 2021; Johnson, 2023; Randall and Spinner, 2023). Clinical trials that focus on quality of life, comorbidity, and survival are necessary to improve survival rates for this expanding population with complex needs.

In HL tissues, few malignant CD30-positive Hodgkin–Reed–Sternberg (H-RS) cells are surrounded by an extended inflammatory stromal reaction comprising heterogeneous, non-neoplastic cell populations, consisting of a broad variety of not only hematologic cells but also mesenchymal cells and a variable degree of fibrosis in the tumor microenvironment (Dürkop et al., 1992; Steidl et al., 2011; Cader et al., 2018; Connors et al., 2020; Ribatti et al., 2022). Recently, we published literature on a less toxic therapy approach, the MEPED schedule, targeting non-oncogene addiction (NOA) targets in HR-S cells and in adjacent, by H-RS cells educated stroma cells with pioglitazone, dexamethasone, everolimus and etoricoxib, as well as low-dose metronomic chemotherapy with treosulfan. MEPED may induce continuous complete remission (cCR) in relapsed/refractory (r/r) HL, irrespective of the kind of pre-treatment used, either chemotherapy, BV, ICPi, autologous, or allogeneic SCT (Ugocsai et al., 2016; Lüke et al., 2021; Pophali et al., 2024; Reuthner et al., 2024).

Taking into account the outstanding results of MEPED in “last-line” therapy and respective modest therapy related side effects, MEPED deserves a detailed discussion in the context of HL pathophysiology, as well as the targeted NOA proteome that copes with HL stress responses and rewires HL hallmarks, thus providing novel perspectives for future HL treatment strategies in the r/r stage (Harrer et al., 2023b).

## MEPED: clinical application

MEPED comprises various orally administered drugs, as depicted in Table 1, that specifically target the tumor microenvironment of HL. Pioglitazone acts to counterbalance HL homeostasis, while the mTOR inhibitor everolimus synergizes with the COX-2 inhibitors etoricoxib and dexamethasone to counteract HL inflammatory responses, which are mostly driven by mTOR. Finally, low-dose metronomic chemotherapy, such as a daily regimen of Treosulfan, is intended to disturb the vascular architecture and communicative network in the tumor microenvironment (Chan et al., 2018; Lüke et al., 2021).

**TABLE 1:** MEPED schedule: concerted transcriptional modulation combined with the induction of stress response.

Drug of MEPED schedule	Dose (mg)	Day application per cycle	Comments
Treosulfan^a^	250	1–28	Mild antiemetic on demand
Pioglitazone^a^	45	1–28	-
Dexamethasone^a^	0.5	1–28	-
Etoricoxib^a^	60	1–28	-
Everolimus^a^	15	1–28	To achieve nadir level of 15 ng/mL

^a^ Scheduled dose reductions.

In the first clinical trial evaluating MEPED in seven patients with refractory or relapsing HL following several lines of pretreatment (third- to sixth-line therapy, including allogeneic stem cell transplantation), a modest toxicity profile and a high rate of complete remissions were observed (Table 2). Moreover, it could be demonstrated that the all-oral MEPED treatment approach for r/r HL is applicable in an outpatient setting irrespective of comorbidities, treatment status, or the sequence of pretreatments. The immunomodulatory feature of MEPED was demonstrated by a significant decrease in C-reactive protein (CRP) in serum during objective response (Ugocsai et al., 2016), as well as a robust neurological improvement in a patient with severe myasthenia gravis who did not qualify for dose-intensive therapy (Fichtner et al., 2020). Regarding MEPED, it is important to emphasize that no single drug component, but the concerted strike of the modular system may induce CR or cCR (Lüke et al., 2021). Case in point, both the entire omission and dose reduction of everolimus below the trough level of 15 μg/L compromised the capacity of MEPED to achieve complete remission, rendering everolimus an essential component of MEPED (Kirchner et al., 2004). To date, several clinical trials have been conducted showing activity of mTOR inhibitors in patients with advanced refractory HL (Table 2). The MEPED schedule has been demonstrated to induce continuous complete remission from the third- to sixth-line therapy, even after the failure of allo-SCT (n = 1) and ASCT (Table 3). CRs occurred regardless of the previous treatment history, including chemotherapy, irradiation, BV, ICI, or autologous or allogeneic SCT, metastatic sites and respective tumor–host interfaces (lung or bone), and disease states (relapsed or refractory) before start of the MEPED schedule (Table 2). The initial intention of MEPED therapy was palliative. Shortening of the adjuvant therapy was possible due to the rapid CR induction within approximately 2–3 months.

**TABLE 2:** Outcome of clinical trials of mTOR inhibition as mono- or combination therapy and a trial with lenalidomide monotherapy in relapsed/refractory Hodgkin’s lymphoma (r/r HL).

• mTOR inhibitor or lenalidomide • mTOR inhibitor combination therapy	Relapsed/refractory	Heavily pretreated	No of patients	ORR	Response	TTP/PFS	Long-term response	Study
Everolimus	+ HL	+	19	47% (95% CI: 24%–71%)	Eight patients achieving a PR and one patient achieving a CR	Median TTP 7.2 months	Four responders remained progression-free at 12 months, one on therapy for 36 months	Johnston et al. (2010)
Everolimus	+ HL	+	57	45.6%	Five patients (8.8%) experienced a complete response, and 21 patients had a partial response (36.8%)	Median PFS 8.0 months (95% CI 5.1–11.0 months)	Seven patients (12%) were long-term responders (2.12 months)	Johnston et al. (2018)
Everolimus + DHAP	+ HL	+	50		CT-based CR rate of 27% (n = 12/45) after two cycles with partial remission of 26 patients (58%)	1-year PFS with everDHAP 83.2%	OS of 90.5% (95% Cl = 73.2–96.9): no improved efficacy versus DHAP	Gillessen et al. (2022)
Temsirolimus/lenalidomide	+ HL	+	20	80%	35% CR	Median PFS of 9.3 months	Median OS of 39.6 months	Major et al. (2022)
Everolimus/lenalidomide	+ NHL/HL	+	55	27%	38% stable disease		-	Padmos et al. (2018)
Lenalidomide	+ HL	+	80	23.8	15% stable disease	Median PFS of 3.7 months	Two patients receiving interrupted lenalidomide had a TTF of 30 and 46 months. Two patients on continuous lenalidomide had long-term response for 24 months (CR) and one PR with 73 months on treatment	Fehniger et al. (2024)
HDAC inhibitors vorinostat/sirolimus (S) or everolimus (E)	+ HL	+	40	55% and 33% (V + S and V + E)	CR in 6 (27%) V + S, CR in 2 (11%) V + E, and PR in 6 (27%) V + S. PR in 4 (22%) V + E	Median PFS of 5.8 months		Janku et al. (2020)
Everolimus, pioglitazone, dexamethasone, etoricoxib, and low-dose metronomic chemotherapy MEPED schedule	+ HL		7	100%	CR in six patients and PR in one patient, with premature discontinuation of study treatment due to availability of haploidentical donor		cCR in all seven patients, with 3 patients without consolidating allogeneic SCT: 126. 127, and 15 months	Reuthner et al. (2024), Lüke et al. (2021)

**TABLE 3:** Clinical data and outcomes of seven relapsed or refractory Hodgkin’s lymphomas.

Patient No	1	2	3	4	5	6	7
Age at diagnosis (years)	55	21	71	27	39	37	57
Sex	Male	Male	Male	Male	Female	Female	Male
Stage at initial diagnosis	IIB	IVA lung	IIIB	IVAE, lung	IVAE, lung	IVB, bone	IIISB, spleen
EBV status, histology	EBV negative nodular sclerosis	EBV-negative	EBV-associated	EBV-negative	EBV-negative	EBV-negative	EBV-negative nodular sclerosis
Additional diseases severely impacting the choice of treatment	No	No	Severe myasthenia gravis	No	No	No	Schizophrenia
Stage at relapse(s) before MEPED	IIIA	IVB	IVB, lung, refractory	IVA	N/A (a), refractory	IVA	IIIA refractory
Lines of therapy before MEPED 1 2 3	BEACOPP/ABVD DHAP + auto-HSCT brentuximab vedotin	BEACOPP DHAP + auto-HSCT	ABVD/AD	BEACOPP DHAP	BEACOPP DHAP + auto-HSCT	BEACOPP DHAP brentuximab vedotin	BEACOPP Brentuximab vedotin ICPi
Irradiation	Paraaortic and pelvic with 27 and 30 Gy	Mediastinal and supraclavicular, 30 Gy	No	Mediastinal, 30 Gy	Mediastinal, 30 Gy	Mediastinal, 36 Gy	No
Previous allo-HSCT	No	Yes	No	No	No	No	No
Previous autologous HSCT	Yes	Yes	No	No	No	Yes	No
Previous ICPi	No	No	No	No	No	No	Yes
Duration of MEPED treatment months	2 residual disease, PR DS-4, after 8 weeks	9 PET DS-2, CR after 24 weeks	14 PET DS-2, CR after 12 weeks	3 PET DS-2, CR after 14 weeks	3 PET DS-2, CR after 14 weeks	10 PET DS-3, CR after 8 weeks	9 PET DS-3, CR after 12 weeks
Delayed add-on of everolimus	No	No	No	Yes	No	No	No
C-reactive protein response	Na	Na	Na	Na	Normalization in CR	Na	Na
Consecutive treatment	allo-HSCT	No	No	allo-HSCT	allo-HSCT	allo-HSCT	No
Outcome	CR cCR 129 months	CR cCR 126 months	CR cCR 127 months myasthenia improved	CR cCR 146 months	CR cCR 151 months	CR cCR 150 months	CR cCR 36 months

## MEPED: rational targeting of NOA in r/r HL

To date, only a few oncogene addiction targets have been identified for the treatment of classic HL, with pulsed chemotherapy remaining the most important element for frontline therapy (Bröckelmann et al., 2023). Next, targeting the tumor immune microenvironment via CD30-targeted immunochemotherapy with brentuximab vedotin (BV) and/or an immune checkpoint inhibitor (ICPi) therapy were a considerable progress to HL therapy (Moskowitz et al., 2021; Fornecker et al., 2023; Herrera et al., 2023; Spinner et al., 2023; Alig et al., 2024). Moreover, targets such as mTOR, COX-2, peroxisome proliferator-activated receptor alpha/gamma (PPARα/γ), and the glucocorticoid receptor have attracted attention as promising avenue to target the inflammatory framework of the HL-related tumor microenvironment (Konopleva et al., 2004; Solimini et al., 2007; Luo et al., 2009; Harrer et al., 2023a; Cuéllar Mendoza et al., 2024). In addition, repetitive pulsed therapies exploit the possibilities to induce comprehensive apoptosis in r/r H-RS cells. Single NOA proteins may even be rate-limiting in HL stress-elicited response pathways, like mTOR. Others are available for re-adjusting pathological homeostatic balances, including nuclear receptor (NR) patterns such as PPARα/γ and the glucocorticoid receptor (Luo et al., 2009; Pierdominici et al., 2017; Chang et al., 2021; Harrer et al., 2023a).

In HL, tumor suppressor genes such as PTEN (Xia et al., 2018) are frequently dysregulated or inactivated, as is growth arrest and DNA damage-inducible protein (GADD45G) (Ying et al., 2005). Phosphatidylinositol 3-kinase/protein kinase B/mammalian target of rapamycin (PI3K/PTEN/AKT/mTOR) signaling appears dysregulated in HL, as it is in many histologically different tumor types (Mossmann et al., 2018). Although, mTOR inhibitors have been used in combined treatment schedules for many tumor types, no comparable long-term treatment results have been achieved (Hua et al., 2019).

The inhibitory function of the oncogenic PI3K/AKT/mTOR signaling network is absent in many tumor types as PTEN is frequently suppressed (Álvarez-Garcia et al., 2019). Pioglitazone may upregulate PTEN in neoplasia (Patel et al., 2001; Teresi and Waite, 2008; Esmaeili et al., 2021).

The resolution of post-therapy developing M-CRAC, i.e., metastases, cancer cell repopulation, acquired resistance, and tumor cell heterogeneity, following pulsed systemic apoptosis-inducing tumor therapies is a critical achievement of modular configured editing approaches targeting neoplastic and stromal cells (Harrer et al., 2023b).

**FIGURE 1:**
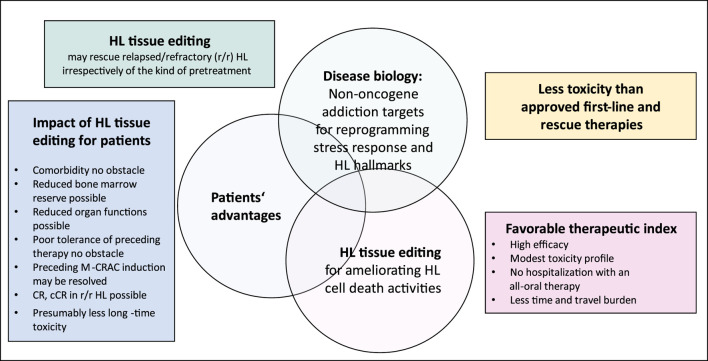
Hodgkin’s lymphoma (HL) tissue editing with MEPED: impact of target selection on patient performance status.

An example of NOA targets distributed within many cell compartments in HL tissue represents the successful targeting of mTOR if edited as therapeutic bottleneck with the MEPED schedule (Figure 1) (Harrer et al., 2023b). ICPs in HL may frequently be targets of oncogene addiction due to the amplification of PD-1 (Gordon et al., 2017; Karihtala et al., 2022).

## MEPED: special role for mTOR inhibition

**FIGURE 2:**
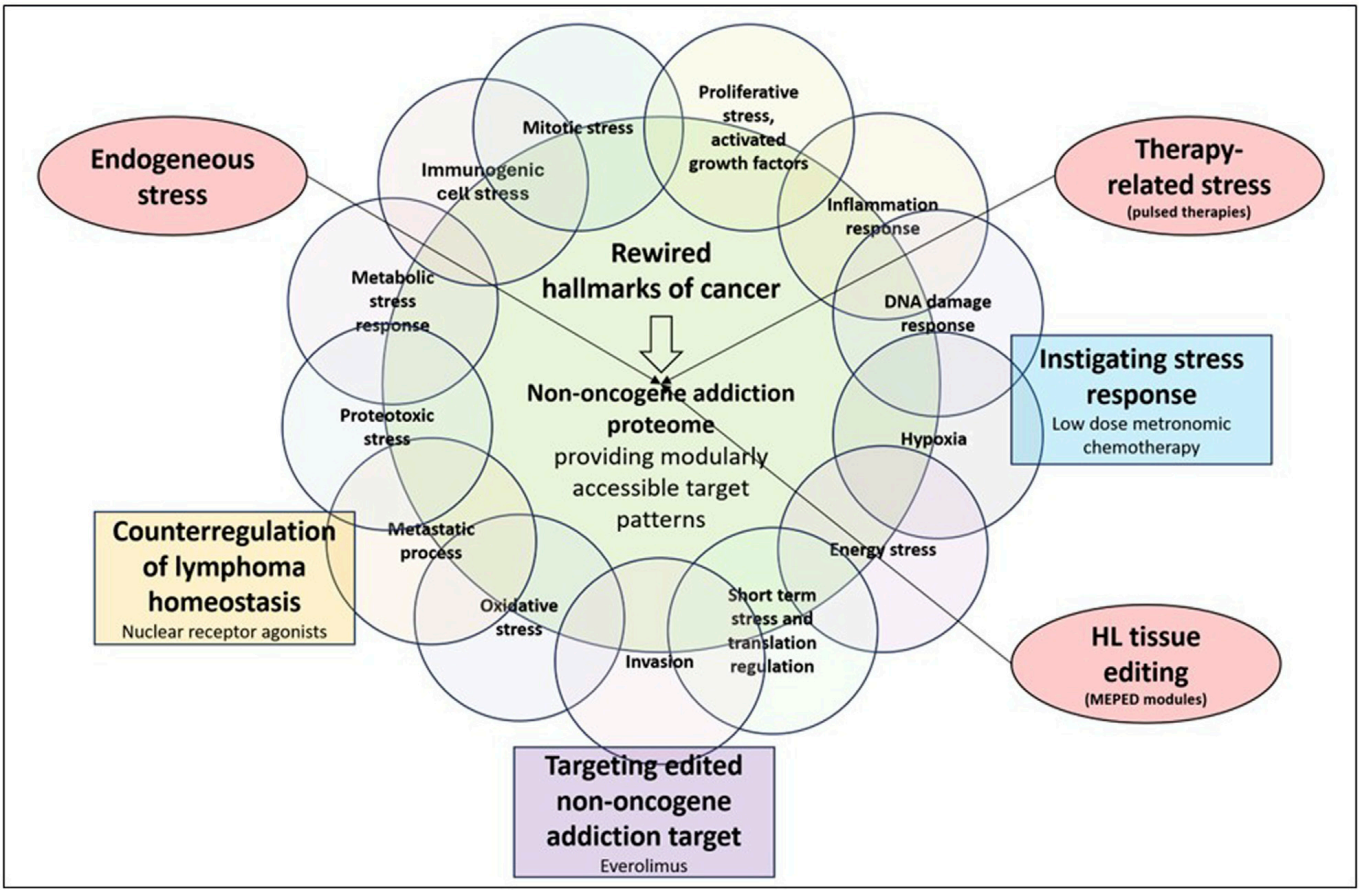
Communicatively integrating, counterregulating, and inhibiting non-oncogene addiction targets, while instigating HL stress response enables durable long-term responses in relapsed/refractory Hodgkin’s lymphoma (r/r HL).

Poor clinical results following mTOR inhibition in r/r HL contrast the pivotal biological role of mTOR in HL stress responses. Activities of HL stress response pathways cumulatively enhance mTOR activity (Figure 2) (Jundt et al., 2005; Márk et al., 2013).

Thus, optimizing the prerequisites for ensuring the efficacy of everolimus by strengthening mTOR addiction in r/r HL is an exemplary issue of how to establish a more efficacious use of inhibitory NOA proteins in the clinical setting.

Relatively few substrates of mTOR contrast with wide-ranging activities on many transcription factors, such as HIF-1α, NF-κB, and signaling pathways, that are involved in r/r HL pathophysiology and are either direct or indirect targets of pioglitazone and dexamethasone, both of which reprogram r/r HL tissue via PI3K, NF-κB, and Wnt pathways, as well as extrinsic and intrinsic apoptotic , STAT, and p53 pathways (Heudobler et al., 2018; Vallée and Lecarpentier, 2018; Rashid et al., 2021; Battaglioni et al., 2022; Glaviano et al., 2023; Harrer et al., 2023a). mTOR activity and constitutive high NF-κB expression are directly correlated in HL (Márk et al., 2013).

Phosphorylation of mTORC1 consistently suppresses multiple autophagy factors (Kim and Guan, 2015). Thus, damaged molecules may not be recycled in response to nutrient starvation (Dossou and Basu, 2019). The availability of nutrients and cellular stress modulate mTORC1 activity. Gain in mTOR and PI3K activity, gain-of-function of AKT, and decreased or lost function of PTEN are common promoters of treatment resistance and disease progression in neoplasia (Glaviano et al., 2023; Harrer et al., 2023b).

## MEPED: therapeutic obstacles while targeting NOA

NOA proteins are undetectable by commonly used genetic and molecular genetic analyses due to only quantitative changes in their expression or altered expression patterns in the case of NRs. Currently, routinely performed front-line diagnostic approaches do not include NOA proteins.

However, if the intended therapies rely on unlocking HL-specific phenotypic characteristics, therapeutic targets derived from the NOA proteome are becoming more prominent and represent a huge, unexplored, modularly linked therapeutic tool that contributes to rewiring communicative interactions while covering the whole HL tissue.

Critical points for targeting NOA include the degree of addiction and the multifunctionality of an NOA target, such as mTOR, the appropriate selection of NR patterns and their selected on-topic activity in communicative networks, potential resistance mechanisms due to escape phenomena, and tumor cell heterogeneity (Harrer et al., 2023b) (Figure 1; Table 3).

mTORC1 plays a central role in activating stress-responsive transcription factors (Aramburu et al., 2014). Therefore, stress response mechanisms, such as those involving NOA proteins, are therapeutically expedient. Elevated levels of reactive oxygen species, as well as spontaneous/therapy-induced DNA damage, ER stress response, and aneuploidy, which are frequently found in HL, cause mTOR adaption to stress responses(Figure 2) (Sen et al., 1990; Bur et al., 2014; Cuceu et al., 2018; Daniel et al., 2019; Alao et al., 2021; Mafi et al., 2023; Marini et al., 2023). Each of these stressors and promoters of mTOR expression/activation correlates with a specific phenotype of cellular stress response (Weigelt et al., 2011). mTORC1 activates stress-responsive transcription factors (Aramburu et al., 2014).

**FIGURE 3:**
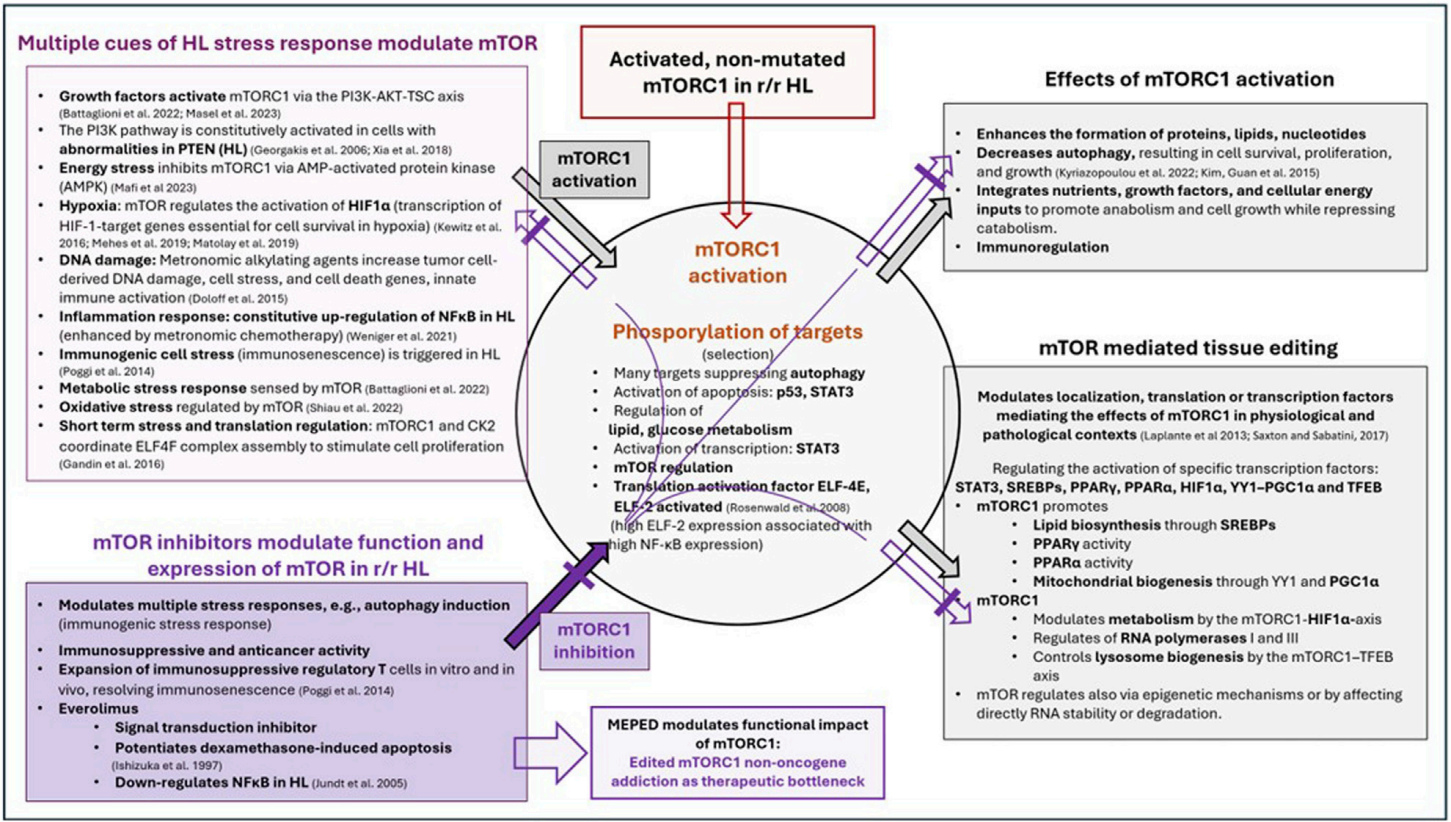
Multiple cues of HL stress response modulate and activate mTOR, followed by mTOR-dependent regulation and activation of specific transcription factors, as well as receptor-triggered transcription factors targeted with the MEPED schedule.

Therefore, the therapeutic potential of mTOR inhibitors is entirely dependent on combinatorial therapeutic strategies while taking into account both stressors related to mTOR induction/activation and the wide range of mTOR effectors (Figure 2). Stress response research revealed that the stressor dose and adaptation time dramatically influence the outcome of in vitro studies (Zelenka et al., 2018). The relationship between mTOR and multiple stressors and effectors characterizes the conserved function of mTOR within stress responses as well as its universal function in a wide range of tumor types, leading to the issue of editing mTOR for strengthening NOA (Figure 3).

The requirements for modulating stressors and effectors of mTOR activity are addressed by the concerted activity profiles of biomodulatory active drugs, as shown in the MEPED schedule (Figure 3) (Ishizuka et al., 1997; Jundt et al., 2005; Georgakis et al., 2006; Rosenwald et al., 2008; Laplante and Sabatini, 2013; Poggi and Zocchi, 2014; Doloff and Waxman, 2015; Kim and Guan, 2015; Gandin et al., 2016; Kewitz et al., 2016; Saxton and Sabatini, 2017; Xia et al., 2018; Matolay et al., 2019; Méhes et al., 2019; Lüke et al., 2021; Weniger and Küppers, 2021; Battaglioni et al., 2022; Kyriazopoulou et al., 2022; Shiau et al., 2022; Mafi et al., 2023; Masel et al., 2023; Reuthner et al., 2024). In contrast, the striking activity profile of MEPED reveals that stress-responsive NOA signaling plays a key role in progressive r/r HL and provides multifold functionally interacting targets for resolving M-CRAC (Harrer et al., 2022; Xu et al., 2024).

## Conclusion

Rescue therapies of r/r HL in the second or third line provide major, yet unresolved problems (Brice et al., 2021). To date, no single, currently available therapy approach for r/r HL >second line may be prioritized (Hazane Leroyer et al., 2022).

Noteworthy developments in targeting oncogenic events in HL, such as using ICPIs, are fraught with difficulties in sufficiently drugging stress response pathways and reprograming HL hallmarks, as systemic pulsed HL therapy may promote M-CRAC via apoptosis induction in r/r HL (Harrer et al., 2022). Instead, the NOA proteome provides a decisive unique modular therapeutic infrastructure to definitely overcome M-CRAC if an HL tissue editing technique is applied.

Tumor tissue editing, along with the MEPED regimen, represents a novel multimodal bioregulatory therapy to treat r/r HL. Targets are NOA mechanisms. The system-directed therapeutic strategy demonstrates potential in achieving complete remission in challenging clinical situations, such as third- to sixth-line therapy.

The presented study results on the metronomic combined application of pioglitazone, dexamethasone, everolimus, etoricoxib, and treosulfan, tested in heavily pre-treated patients, and the resulting high rate of CRs (six of seven patients) and seven cCRs made us to study the mechanistic activity profile of everolimus using available clinical data on everolimus in r/r HL and the huge amount of experimental data on stress responses and mTOR activity in HL (Figure 3). The exploration provides a detailed explanation of how a multimodal HL tissue editing therapy targets r/r HL cells and the adjacent microenvironment, highlighting mTOR as a critical therapeutic bottleneck due to concerted transcription modulation and MEPED as a regulator of HL stress responses and HL hallmarks.

The pilot study on MEPED requires definitive validation in a larger multicenter trial because it lacks comprehensive statistical power and raises potential concerns, particularly whether the results may be generalized across all HL subgroups and whether responders may be selected via molecular profiling. Long-term sustainability of complete remission in r/r HL (seven patients) stimulates to proceed with further validating studies (Table 2).

The MEPED approach represents a promising therapeutic strategy for r/r HL, with significant potential in challenging clinical scenarios. The research requires, however, further investigation to establish its definitive clinical utility. If validated, this approach could provide a less toxic, more targeted treatment option for patients with advanced r/r HL, thereby improving both survival and quality of life.
